# *Wolbachia* Utilizes Host Microtubules and Dynein for Anterior Localization in the *Drosophila* Oocyte

**DOI:** 10.1371/journal.ppat.0010014

**Published:** 2005-10-14

**Authors:** Patrick M Ferree, Horacio M Frydman, Jennifer M Li, Jian Cao, Eric Wieschaus, William Sullivan

**Affiliations:** 1 Department of Molecular, Cellular, and Developmental Biology, University of California, Santa Cruz, California, United States of America; 2 Howard Hughes Medical Institute, Department of Molecular Biology, Princeton University, Princeton, New Jersey, United States of America; Stanford University, United States of America

## Abstract

To investigate the role of the host cytoskeleton in the maternal transmission of the endoparasitic bacteria *Wolbachia,* we have characterized their distribution in the female germ line of *Drosophila melanogaster*. In the germarium, *Wolbachia* are distributed to all germ cells of the cyst, establishing an early infection in the cell destined to become the oocyte. During mid-oogenesis, *Wolbachia* exhibit a distinct concentration between the anterior cortex and the nucleus in the oocyte, where many bacteria appear to contact the nuclear envelope. Following programmed rearrangement of the microtubule network, *Wolbachia* dissociate from this anterior position and become dispersed throughout the oocyte. This localization pattern is distinct from mitochondria and all known axis determinants. Manipulation of microtubules and cytoplasmic Dynein and Dynactin, but not Kinesin-1, disrupts anterior bacterial localization in the oocyte. In live egg chambers, *Wolbachia* exhibit movement in nurse cells but not in the oocyte, suggesting that the bacteria are anchored by host factors. In addition, we identify mid-oogenesis as a period in the life cycle of *Wolbachia* in which bacterial replication occurs. Total bacterial counts show that *Wolbachia* increase at a significantly higher rate in the oocyte than in the average nurse cell, and that normal *Wolbachia* levels in the oocyte depend on microtubules. These findings demonstrate that *Wolbachia* utilize the host microtubule network and associated proteins for their subcellular localization in the *Drosophila* oocyte. These interactions may also play a role in bacterial motility and replication, ultimately leading to the bacteria's efficient maternal transmission.

## Synopsis

Intracellular bacteria that cause disease exploit host cells for their survival and reproduction. Here the authors are studying *Wolbachia,* an intracellular bacteria that infects many insect species and other invertebrates, such as filarial nematodes, the agents of diseases such as African river blindness and elephantiasis. *Wolbachia* transmission is similar to mitochondrial inheritance, that is, from mother to offspring in the host. However, little is known about the mechanisms of this maternal transmission and the interactions of *Wolbachia* and its hosts at the cellular level. Here the authors show that in the fruit fly *Drosophila melanogaster, Wolbachia* bacteria are initially distributed uniformly throughout the female germ line but concentrate during the middle stages of oogenesis in the future oocyte, exhibiting a striking anterior localization that is distinct from mitochondria and all known axis determinants. The authors demonstrate that microtubules and the minus-end-directed motor Dynein are required for this subcellular localization, suggesting that *Wolbachia* uses the host's microtubule cytoskeleton and transport system to ensure its transmission to the next generation. This microtubule dependence contrasts with the Actin-based motility observed for many other intracellular bacteria and may provide a foundation for further probing of the molecular and cellular basis of *Wolbachia* replication and transmission.

## Introduction

Associations between bacterial endoparasites and their hosts are widespread in nature [1]. Studies of how these endoparasites modify host cell biological processes have proven insightful in elucidating the mechanisms that drive core cellular events [2]. While interactions between intracellular pathogens and the host Actin cytoskeleton have been intensively investigated [3], interactions between pathogens and the microtubule cytoskeleton are less well explored. In insects, the endoparasitic bacteria *Wolbachia* maintains a close association with host microtubules [4,5], thus providing an excellent system to explore pathogen interactions with the microtubule cytoskeleton.

*Wolbachia* are gram-negative, *Rickettsia*-like bacteria that infect insects (reviewed in [6]), filarial nematodes (reviewed in [7]), crustaceans [8], and mites [9]. These bacteria are most prominent in insects, infecting an estimated 16% to 76% of insect species worldwide [10–12]. This is largely attributable to their ability to manipulate the host insect reproductive machinery for their own benefit. Depending on the insect species and bacterial strain, the presence of *Wolbachia* in females can induce parthenogenesis [13], male killing [14], or feminization of male progeny [15], all of which increase the number of infected females. The most common *Wolbachia*-induced host manipulation is a form of reproductive sterility called cytoplasmic incompatibility (reviewed in [6,16]). Cytoplasmic incompatibility occurs when an infected male mates with an uninfected female, resulting in high levels of embryonic death. Embryonic development is normal, however, if the female is infected with the same *Wolbachia* strain. Because infected females can successfully mate with either infected or uninfected males, they are selectively favored over uninfected females. *Wolbachia* are maternally transmitted, so favoring infected females enhances bacterial transmission frequencies.

Although *Wolbachia* have been detected in host somatic tissues, they are found primarily in the germ-line tissues [17–20]. In males, *Wolbachia* are present within developing spermatocytes, but they are completely removed in cytoplasmic “waste bags” during late spermatogenesis [19,21]. Because *Wolbachia* are not transmitted through the sperm, their presence in males is considered to be a “dead end” with regard to transmission [22,23]. *Wolbachia* are instead transmitted from the mother to her eggs, where they persist through embryogenesis and eventually become incorporated into the pole cells, the precursors of germ-line stem cells [5]. *Wolbachia* transmission frequencies previously have been measured to be 100% from laboratory-reared *Drosophila melanogaster* and *D. simulans* females [24] and over 97% from field-collected *D. melanogaster* females [25], indicating that *Wolbachia* are capable of infecting developing eggs with nearly perfect fidelity in these species. As only minor reductions in fecundity have been observed for infected females [26], transmission of *Wolbachia* involves their amplification in the female germ line to levels high enough to insure infection of nearly all eggs while minimizing disruption of normal oocyte development [22]. Little is known about the cellular mechanisms underlying the efficient maternal transmission of *Wolbachia*.

**Figure 1:**
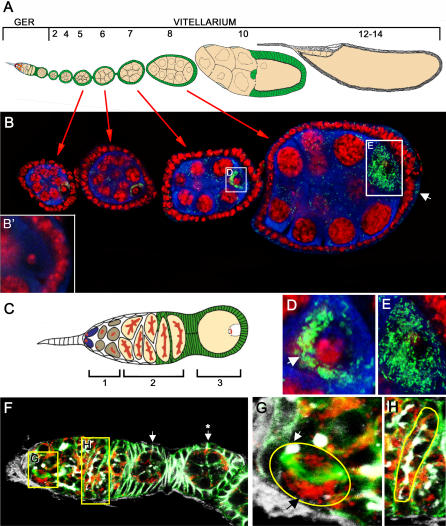
*Wolbachia* Distribution from Germ-Line Stem Cell Division through Mid-Oogenesis In (B), (B′), (D), and (E), *Wolbachia* is green (hsp60), Vasa is blue, and DNA is red. In (F–H), *Wolbachia* is red (hsp60), α-Spectrin highlights the fusome (white), and microtubules are green. (A) The ovariole consists of the germarium (GER) at the anterior-most tip, and the vitellarium. Numbers represent the stages of oogenesis in the vitellarium (stages 2 through 14). Between stages 2 and 7, the cytoplasm of the nurse cells and the oocyte increases linearly. Following stage 7, the oocyte cytoplasm begins to grow disproportionately faster than nurse cell cytoplasm. Between stages 10 and 12, all nurse cell cytoplasm is transferred into the oocyte through “cytoplasmic dumping.” From stage 12 to 14, the nurse cell nuclei degenerate and dorsal filaments are formed. (B) Middle stage egg chambers infected with *Wolbachia*. White arrow indicates *Wolbachia* within follicle cells. (B′) High magnification of a stage 5 uninfected oocyte stained with anti-hsp60 antibody, showing that this antibody does not cross react with *D. melanogaster* proteins. (C) The germarium is divided into regions 1, 2, and 3. In the germarium, a germ-line stem cell (dark blue) divides to produce a daughter stem cell and a cystoblast. In region 1, the cystoblast is mitotically active; it undergoes four successive rounds of mitosis, resulting progressively in cysts of two, four, eight (brown), and sixteen (tan) cystocytes. Red shading represents the fusome. In region 2, the 16-cell cyst becomes surrounded by somatically derived follicle cells (green). At the most posterior position of the germarium (region 3), the stage 1 cyst buds off and enters the vitellarium. By this time the oocyte (white) has moved to the posterior side of the cyst. (D) High magnification of a stage 6 oocyte. *Wolbachia* (indicated by a white arrow) accumulate densely around the anterior of the oocyte. (E) High magnification of a stage 7 oocyte. *Wolbachia* begin to disperse from the anterior of the oocyte. (F) A germarium infected with *Wolbachia*: stage 1 egg chamber (white arrow) and stage 2 egg chamber (white arrow with asterisk). (G) High magnification of a germ-line stem cell (circumscribed by yellow line) undergoing asymmetric mitosis. The fusome material (indicated by a white arrow) is spherical in the stem cell. A cystoblast is visible in the upper right corner, identified by its shape and position in the germarium. A black arrow indicates *Wolbachia*. (H) A 16-cell cyst in region 2 of the germarium (circumscribed by yellow line) as it becomes surrounded by follicle cells. *Wolbachia* are present in all of the germ cells, but do not associate with the fusome. (A) adapted from [79]; (C) adapted from [39].

The microtubule cytoskeleton and associated factors play a large role in proper oocyte formation. In *D. melanogaster,* oocytes develop from one of several germ-line stem cells located at the anterior tip of the ovary (see Figure 1; for reviews of *Drosophila* oogenesis, see [27,28]). Each stem cell divides asymmetrically to produce a daughter stem cell and a cystoblast. The cystoblast undergoes four synchronized mitotic divisions to form a cluster, or cyst, of 16 germ cells termed cystocytes. These mitotic divisions occur with incomplete cytokinesis so that the cystocytes remain interconnected by cytoplasmic bridges known as ring canals. Each of the initial two cystocytes derived from the first mitotic division contain four ring canals, while the other cystocytes have three or fewer. Despite the fact that they share a common cytoplasm, one of the two cystocytes with four ring canals differentiates into the oocyte, while the other 15 become supporting trophocytes, termed “nurse” cells. Differentiation of the oocyte and its specialized development throughout oogenesis require the activities of specific cytoskeletal components, such as the Actin- and Spectrin-containing fusome and different forms of an organized microtubule network [29]. During mitotic division of the germ cells, the fusome spans the ring canals of all the germ cells but concentrates more in one of the two cells containing four ring canals [30,31]. There, its presence is necessary for the establishment of a microtubule network nucleated within this particular germ cell. The plus ends of these microtubules project through the ring canals and into the nurse cells and facilitate minus-end-directed transport of specific mRNA and proteins, leading to differentiation of this cell into the oocyte [32]. Although the fusome degenerates shortly after oocyte differentiation, the polarized microtubule network continues to direct further transport of other maternal factors produced in the nurse cells into the oocyte until the end of mid-oogenesis [33,34]. At this time, the microtubule network reverses polarity [35], and transported maternal factors are then placed in specific regions within the oocyte with the aid of the microtubule-associated motors Dynein and Kinesin (reviewed in [36]).

In this study, we address the role(s) of the host cytoskeleton in *Wolbachia*'s transmission from germ-line stem cell to mature oocyte in *D. melanogaster*. In the germarium, *Wolbachia* are distributed among all germ cells of the cyst, establishing an early oocyte infection. During early oogenesis, *Wolbachia* show a distinct localization at the anterior end of the oocyte, where many bacteria appear to contact the anterior side of the nuclear envelope. Following programmed rearrangement of the microtubule network, *Wolbachia* dissociate from this anterior position and become evenly dispersed throughout the oocyte. This localization pattern is distinct from mitochondria and all known axis determinants. Manipulation of microtubules and cytoplasmic Dynein and Dynactin, but not Kinesin-1, disrupts anterior bacterial localization in the oocyte. In live egg chambers, *Wolbachia* exhibit movement in nurse cells but not in the oocyte, suggesting that the bacteria are anchored by host factors. In addition, we find that the number of *Wolbachia* in the oocyte increases at a higher rate than bacteria in the average nurse cell. These findings demonstrate that *Wolbachia* utilize the host microtubule network and associated proteins for their subcellular localization in the oocyte. These interactions may also play a role in bacterial motility and replication, ultimately leading to their efficient maternal transmission.

## Results

### *Wolbachia* Become Distributed throughout the Cyst Prior to Oocyte Differentiation

A diagram of oogenesis is presented in Figure 1A. The production of a functional oocyte begins with the division of a stem cell in region 1 of the germarium, the anterior-most structure of the ovariole (Figure 1A and 1C). Each stem cell division gives rise to a daughter stem cell and a cystoblast. The cystoblast then undergoes four rounds of mitosis with incomplete cytokinesis to produce a cyst of 16 germ cells, termed cystocytes, which are interconnected by cytoplasmic bridges. To determine the distribution of the *Wolbachia* strain *w*Mel during these divisions in *D. melanogaster,* we stained ovarioles with an antibody against recombinant human heat shock protein 60 (hsp60), which recognizes the bacterial homolog [24,37,38] but does not cross-react with *Drosophila* proteins (Figure 1B). In the germarium, *Wolbachia* were seen in dividing stem cells (Figure 1F and 1G), as well as in all 2-, 4-, 8-, and 16-cell cysts (Figure 1F and 1H), indicating that the bacteria are efficiently passed from the stem cell to each of the interconnected germ cells of the cyst. By distributing evenly among all 16 germ cells prior to oocyte differentiation, *Wolbachia* establish an early presence in the cell destined to become the oocyte.

In region 2 of the germarium, one of the two germ cells with four ring canals differentiates into the oocyte, while the remaining 15 germ cells become nurse cells. In addition to its involvement in oocyte differentiation [31,32], the fusome plays a role in the partitioning of organelles among the dividing germ cells in the germarium [39]. To determine whether *Wolbachia* localize with the fusome during germ cell division, we stained the bacteria with anti-hsp60 and the fusome with anti-α-Spectrin (Figure 1F–1H). In contrast to mitochondria [39], *Wolbachia* do not associate with the fusome in the dividing stem cells or 16-cell cysts, but instead they appear distributed within the cytoplasm of the germ cells (Figure 1G and 1H). This observation suggests that *Wolbachia* and mitochondria have different modes of segregation among the dividing germ cells.

### During Mid-Oogenesis, *Wolbachia* Concentrate Anteriorly in the Oocyte

Following cyst formation, the oocyte moves posteriorly within the cyst as the cyst moves into the posterior-most position of the germarium. The cyst, now termed “egg chamber,” has reached region 3 (also referred to as stage 1) and becomes completely surrounded by somatically derived follicle cells (Figure 1C). From this stage until stage 7 in the vitellarium, the nurse cell nuclei increase over 100-fold in volume as they undergo 10–12 rounds of endoreplication [40], while the oocyte nucleus arrests in prophase of meiosis I. Despite the difference in nuclear volume between the nurse cells and the oocyte, the cytoplasmic volumes of the nurse cells and oocyte increase at the same rate and are nearly equal through stage 7 [41].

**Figure 2:**
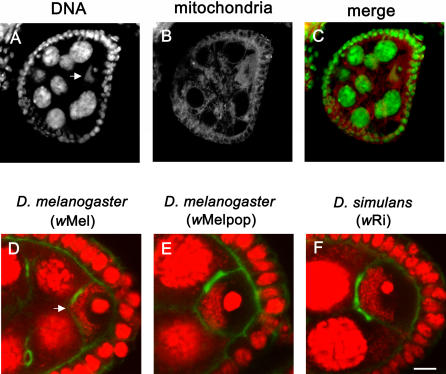
*Wolbachia* and Mitochondria Exhibit Different Localizations, and *Wolbachia* Anterior Localization Is Observed for Several Bacterial Strains and *Drosophila* Species (A–C) A stage 5 egg chamber showing *Wolbachia* and mitochondria. (A) Host DNA and *Wolbachia* (greyscale-OliGreen). (B) Mitochondria (greyscale-MitoTracker). (C) Merged image of (A) and (B): *Wolbachia* and host DNA are green and Mitochondria are red. (D–F) *Wolbachia* and host DNA are red (propidium iodide), and cell cortex and ring canals are green. (D) An oocyte from *D. melanogaster* infected with the *w*Mel *Wolbachia* strain. (E) An oocyte from *D. melanogaster* infected with the *w*Melpop (Popcorn) *Wolbachia* strain. (F) An oocyte from *D. simulans* infected with the *w*Riv *Wolbachia* strain. Scale bar = 10 μm. White arrows in (A) and (D) indicate *Wolbachia* concentrated at the anterior region of the oocyte.

During stages 1 and 2, *Wolbachia* in the nurse cells and oocyte were evenly distributed within cell cytoplasm and showed similar densities in these cell types (Figure 1F). In nurse cells, *Wolbachia* remained distributed throughout the cytoplasm for the remainder of oogenesis (Figure 1B). However, in the oocyte, *Wolbachia* began to concentrate between the anterior cortex and the nucleus around stage 3 (Figure 1B). By stage 6, the anterior concentration of *Wolbachia* became most pronounced, as they collectively formed a “cup” surrounding the anterior periphery of the nucleus (Figure 1D). Consequently, at this point in oogenesis, many bacteria in the oocyte were proximal to the oocyte nucleus. In contrast, mitochondria were distributed throughout the oocyte and nurse cell cytoplasm (Figure 2A–2C).

All egg chambers from infected females contained *Wolbachia,* but occasionally we observed uninfected nurse cells or oocytes even though adjacent cells were infected at normal levels. Of 35 stage 4–7 oocytes from infected egg chambers, four were devoid of *Wolbachia* (not shown). The lack of bacteria in these germ cells was likely not due to developmental defects, as they appeared normal in morphology. *Wolbachia* densities were lower in the follicle cells that surround the egg chamber than in the germ cells (see Figure 1B).

To determine whether this bacterial localization pattern is present in other *Drosophila* species and *Wolbachia* strains, we also examined *Wolbachia*'s distribution in egg chambers from *D. simulans* infected with *w*Ri [42] and *D. melanogaster* infected with *w*Melpop (“Popcorn”) [43] (Figure 2). In both cases, *Wolbachia* exhibited a prominent anterior localization in stage 5–7 oocytes (Figure 2E and 2F), suggesting that this pattern is common among these *Drosophila* species and *Wolbachia* strains.

### *Wolbachia* Exhibit Movement in Nurse Cells but Not in the Oocyte In Vivo

**Figure 3:**
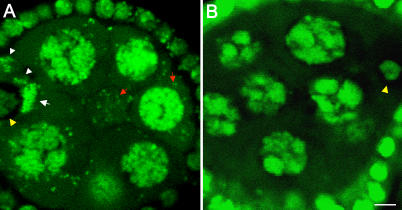
*Wolbachia* Exhibit Movement in Nurse Cells but Not in the Oocyte In Vivo (A) A still image taken from Video S1 showing bacterial motility in a live stage 5 egg chamber. Draq 5 stains *Wolbachia* and host DNA (both shown in green). As in fixed tissues, *Wolbachia* localize anteriorly (white arrow) in the oocyte and are distributed randomly in the nurse cells (red arrows) of live egg chambers. *Wolbachia* in nurse cells exhibit random movement while *Wolbachia* in the oocyte are relatively motionless (Video S1). White arrowheads indicate smaller groups of *Wolbachia* at the anterior region of the oocyte with lower bacterial densities, suggesting that their lack of movement is not due to bacterial overcrowding in the oocyte. Yellow arrowhead indicates the oocyte nucleus. (B) A still image taken from Video S2 showing a live stage 6 uninfected egg chamber. This image illustrates that Draq 5 stains *Wolbachia* (see [A]) but not mitochondria or other cytoplasmic organelles. Yellow arrowhead indicates the oocyte nucleus. Scale bar = 5 μm.

To further investigate the behavior of *Wolbachia* in the oocyte, we visualized the bacteria in vivo using the DNA dye Draq 5 (see Materials and Methods). In live egg chambers, *Wolbachia* localized anteriorly in the oocyte but were randomly distributed within nurse cell cytoplasm, confirming our observations from fixed samples (Figure 3A; Video S1). Stainings with control uninfected egg chambers confirmed that Draq 5 highlights host DNA but not mitochondria or other cytoplasmic organelles (Figure 3B; Video S2).

Additionally, *Wolbachia* exhibited random movement within the nurse cells, whereas in the oocyte, they appeared nearly motionless (Video S1). Occasionally, small clusters of bacteria could be seen adjacent to areas within the oocyte cytoplasm devoid of other bacteria (Figure 3A; Video S1), suggesting that the lack of bacterial movement in the oocyte is not a result of overcrowding. These observations suggest that some difference in subcellular environment leads to *Wolbachia* movement in nurse cells and a lack of their movement in the oocyte.

### *Wolbachia* Preferentially Concentrate in the Oocyte during Stages 3–7

**Figure 4:**
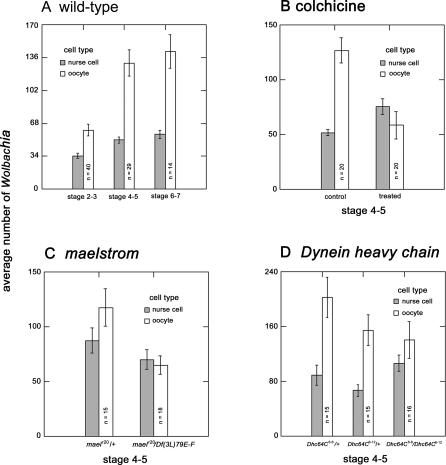
Manipulation of Specific Host Cytoskeletal Factors Affects *Wolbachia* Numbers (A) Mean *Wolbachia* numbers for the average nurse cell and the oocyte through mid-oogenesis. (B) Mean *Wolbachia* numbers for the average nurse cell and the oocyte for colchicine-treated and control (untreated) egg chambers. (C) Mean *Wolbachia* numbers for the average nurse cell and the oocyte for *maelstrom* ^r20^/+ and *maelstrom* ^r20^/*Df (3L) 79E-F* egg chambers. (D) Mean *Wolbachia* numbers for the average nurse cell and the oocyte for *Dhc64C* ^6–6^/+, *Dhc64C* ^6–12^/+, and *Dhc64C* ^6–6^/*Dhc64C* ^6–12^ egg chambers. Error bars represent the standard error of the mean.

We observed that *Wolbachia* numbers appeared to increase disproportionately faster in the oocyte relative to the nurse cells during stages 3–7. To verify and quantify this observation, for each egg chamber the total number of *Wolbachia* in the oocyte was determined, as well as the average from counts of total *Wolbachia* in four randomly chosen nurse cells (Figure 4). Total cell counts were obtained through confocal serial sectioning of formaldehyde-fixed egg chambers stained with propidium iodide (see Materials and Methods). During these stages fixed cytology is excellent for accurate scoring of total *Wolbachia* numbers because of small cell volume. However, accurate bacterial counts beyond stage 7 were not possible because of the increased volume and yolkiness of the oocyte cytoplasm. The mean total number of *Wolbachia* in the oocyte from stages 2–3 to stages 6–7 increased from 61.0 (standard error of the mean, ± 6.1) to 142.3 (± 17.3), while the mean total number of *Wolbachia* in the average nurse cell during this period increased from 34.4 (± 2.6) to 56.5 (± 4.5) (Figure 4A). Two-way ANOVA showed an overall effect of cell type on the number of *Wolbachia* (F_1,126_ = 78.5, *p* < 0.0001), but with a significant interaction between cell type and stage (F_2,126_ = 7.6, *p* < 0.001). A Tukey post-hoc test verified that there were significantly more *Wolbachia* in the oocyte than in the average nurse cell for all stages. However, between stages 2–3 and 6–7 *Wolbachia* numbers increased more dramatically in the oocyte than in the average nurse cell (230% versus 160%, respectively), as reflected by the significant interaction term.

### Loss of *Wolbachia*'s Anterior Localization Coincides with Microtubule Reorganization in the Oocyte

**Figure 5:**
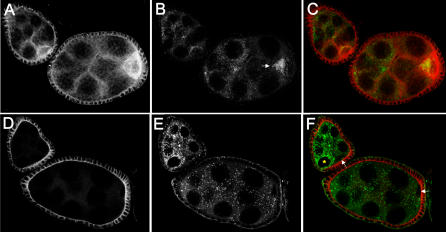
Depolymerization of Microtubules Causes Loss of Anterior *Wolbachia* Localization in the Oocyte (A–C) Stage 5 and 7 infected egg chambers, untreated. (A) Microtubules (greyscale). (B) *Wolbachia* (greyscale). White arrow indicates *Wolbachia* concentrated at the anterior region of the oocyte. (C) Merged image of (A) and (B): microtubules are red, and *Wolbachia* are green (hsp60). (D–F) Stage 5 and 7 infected egg chambers treated with colchicine. (D) Microtubules (greyscale). (E) *Wolbachia* (greyscale). (F) Merged image of (D) and (E): microtubules are red, and *Wolbachia* are green (hsp60). White arrows indicate positions of the oocytes. Yellow asterisk indicates a nurse cell adjacent to the oocyte in which *Wolbachia* appear abnormally high.

Through stage 6, the microtubule-organizing center (MTOC) of the egg chamber resides at the posterior pole of the oocyte and the plus ends of the microtubules extend through the ring canals and into the nurse cells [44]. These microtubules facilitate the inward transport of maternal mRNAs and proteins necessary for proper oocyte and embryonic development [44]. However, between stages 7 and 8 the MTOC disassembles and microtubules become nucleated from the anterolateral cortex of the oocyte [35]. Before stage 8, *Wolbachia* maintained a tight anterior concentration, but after stage 8 they became dispersed throughout the entire oocyte (compare Figure 1D to 1E). To confirm the correlation between *Wolbachia* redistribution and microtubule reorganization, we double-stained infected *D. melanogaster* oocytes with anti-α-Tubulin antibodies to label the microtubules and anti-hsp60 to label *Wolbachia*. The images in Figure 5 demonstrate that prior to stage 8, *Wolbachia* concentrated opposite the MTOC located at a position near the posterior pole. However, *Wolbachia* began to lose their anterior localization in many oocytes around stage 8. In nearly all stage 9 oocytes, *Wolbachia* had become dispersed throughout the oocyte cytoplasm, although a few oocytes exhibited a slight residual accumulation of *Wolbachia* at the anterior region. *Wolbachia* were completely dispersed in all stage 10 oocytes. The correlation between timing of microtubule reorganization and loss of anterior *Wolbachia* localization suggests that the bacteria rely on microtubules for positioning within the oocyte.

### Cytoplasmic Dumping Results in the Transfer of *Wolbachia* from Nurse Cell Cytoplasm into the Oocyte

**Figure 6:**
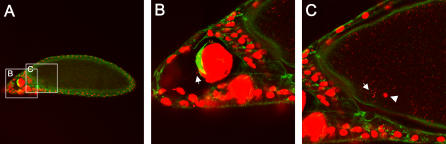
*Wolbachia* Are Transferred into the Oocyte from Nurse Cells through Cytoplasmic Dumping *Wolbachia* and host DNA are red (propidium iodide), and cell cortex and ring canals are green. (A) A stage 12 egg chamber nearing completion of cytoplasmic dumping. (B) Higher magnification of nurse cell remnants. Only a small number of *Wolbachia* are present in remaining nurse cell cytoplasm. White arrow indicates a few *Wolbachia* near a nurse cell nucleus. (C) Higher magnification of the anterior region of the oocyte. Nearly all *Wolbachia* have been “dumped” into the oocyte. White arrow indicates *Wolbachia,* and white arrowhead points to the oocyte nucleus.

At stage 10, the nurse cell cytoplasm is transferred through the ring canals into the oocyte. This Actin/Myosin-based process, termed “cytoplasmic dumping,” occurs within a relatively short period of 30 min and is completed by stage 12 [45–47]. Just before cytoplasmic dumping initiates, *Wolbachia* were abundant in the nurse cells (not shown). However, by stage 12 virtually all *Wolbachia* resided in the oocyte, with only a few bacteria remaining in the nurse cell remnants (Figure 6). This suggests that during cytoplasmic dumping *Wolbachia* are effectively delivered to the oocyte with nurse cell cytoplasm.

### Microtubules Are Required for the Anterior Localization of *Wolbachia* in the Oocyte

The fact that *Wolbachia*'s distribution within the oocyte changes following the developmentally programmed microtubule reorganization led us to speculate that *Wolbachia* localization is microtubule dependent. We tested this idea by analyzing the distribution of *Wolbachia* in oocytes from females that were fed colchicine, a microtubule inhibitor. Treatment with colchicine resulted in complete depolymerization of microtubules within the germ cells, but not in follicle cells, where microtubules are more stable (see Figure 5D and 5F). Following colchicine treatment, *Wolbachia* failed to localize at the anterior of the oocyte (Figure 5E and 5F). Additionally, in some treated egg chambers, the number of *Wolbachia* appeared to increase within nurse cells adjacent to the oocyte (Figure 5E and 5F). *Wolbachia* localization does not appear to depend on the Actin cytoskeleton, as exposure to the filamentous Actin depolymerizer, cytochalasin-D, did not affect their localization within the oocyte (not shown).

**Figure 7:**
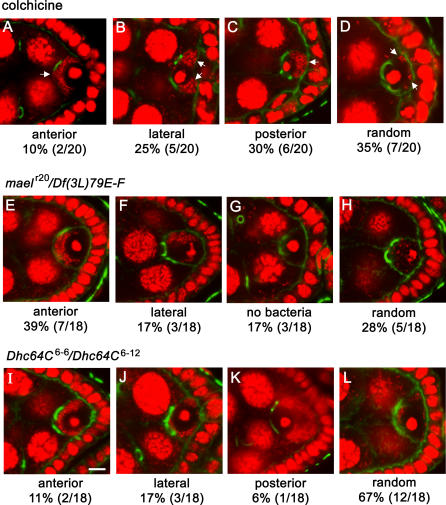
Perturbation of Microtubules and Cytoplasmic Dynein Disrupts Anterior Localization of *Wolbachia* in the Oocyte *Wolbachia* and host DNA are red (propidium iodide), and cell cortex and ring canals are green. (A–D) Stage 4–6 infected oocytes from colchicine-treated females. White arrows indicate *Wolbachia*. (E–H) Stage 4–6 infected oocytes from *maelstrom* ^r20^/*Df (3L) 79E-F* females. (I–L) Stage 4–6 infected oocytes from *Dhc64C* ^6–6^/*Dhc64C* ^6–12^ females.

To further investigate how the disruption of microtubules affects *Wolbachia* localization in the oocyte, we classified a number of colchicine-treated oocytes based on their bacterial localization pattern. Of 20 stage 4–6 colchicine-treated oocytes, only two exhibited normal anterior localization (Figure 7A). Six exhibited posterior localization, 11 exhibited lateral and posterior cortical localization, and in seven oocytes, *Wolbachia* were dispersed randomly throughout the cytoplasm (Figure 7B–7D). In contrast, of 31 control (untreated) oocytes infected with *Wolbachia,* all exhibited anterior bacterial localization. The distribution of *Wolbachia* in the nurse cells appeared unaffected by colchicine treatment (not shown).

The role of microtubules in *Wolbachia*'s localization was further tested by examining bacterial distribution in oocytes derived from *maelstrom* ^r20^/*Df (3L) 79E-F* females. In *maelstrom* mutants, the microtubule minus ends are focused aberrantly on the anterior side of the nucleus, and specific axis determinants become misplaced because of an abnormal microtubule polarity [48]. Of 18 stage 4–6 *maelstrom*-derived oocytes, 11 showed dramatic mislocalization of *Wolbachia* (Figure 7E–7H). The range of defects was similar to those observed in colchicine-treated oocytes. Three of 11 oocytes exhibited lateral localization, and in five oocytes *Wolbachia* were randomly dispersed throughout the cytoplasm. *Wolbachia* were absent from three oocytes. Taken together, these results support the idea that *Wolbachia* utilize microtubules for normal anterior localization in the oocyte.

### Cytoplasmic Dynein and Dynactin, but Not Kinesin-1, Are Required for Anterior Localization of *Wolbachia* in the Oocyte

The localization of *Wolbachia* at the anterior of the oocyte during stages 3–7 is opposite to the site of microtubule nucleation, which is positioned at the posterior pole at this time. Therefore, we hypothesized that *Wolbachia* may associate with the microtubule plus-end-directed motor, Kinesin-1, for early anterior localization. To test this idea, we used the FLP-FRT system (see Materials and Methods) to generate females whose germ line is completely devoid of the Kinesin heavy chain (Khc). *Wolbachia* localized normally in all oocytes derived from *Khc* ^27^ null females (not shown), indicating that *Wolbachia* do not require Kinesin-1 for anterior positioning.

To test whether *Wolbachia*'s anterior localization instead requires the microtubule minus-end-directed motor, cytoplasmic Dynein, we visualized *Wolbachia* in oocytes derived from infected females transheterozygous for two hypomorphic alleles of the Dynein heavy chain (Dhc), *Dhc64C* ^6–6^ and *Dhc64C* ^6–12^. This combination of alleles results in abnormal patches of Dynein in nurse cells, misplacement of Dynein in the oocyte, and female sterility, without disrupting the microtubule network [49]. In oocytes from *Dhc64C* transheterozygous females, *Wolbachia* displayed a variety of abnormal distribution patterns (Figure 7I–7L). Of 18 stage 4–6 *Dhc64C*-derived oocytes, only two exhibited normal anterior localization. Three oocytes exhibited laterally distributed *Wolbachia,* one showed posterior-localized *Wolbachia,* and 12 exhibited *Wolbachia* that were distributed randomly throughout the cytoplasm.

**Figure 8:**
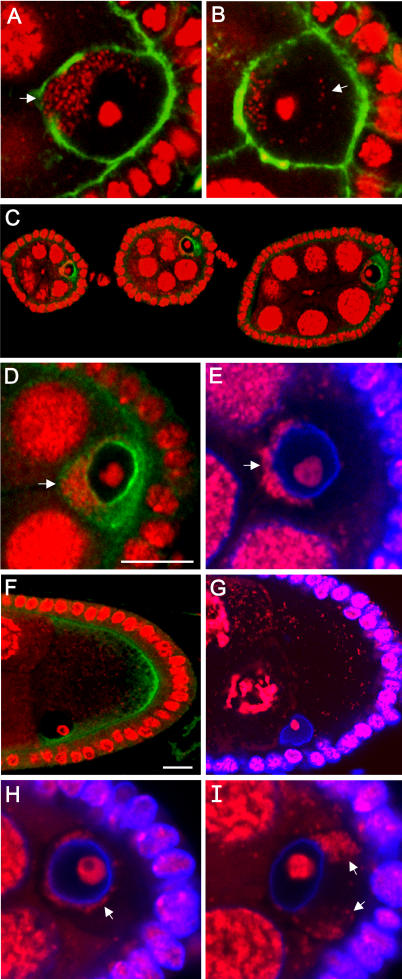
In the Oocyte, *Wolbachia* Localize along the Anterior Nuclear Envelope In (A) and (B), *Wolbachia* and host DNA are red (propidium iodide), and cell cortex and ring canals are green. In (C), (D), and (F),) cytoplasmic Dynein is green, and *Wolbachia* and host DNA are red (OliGreen). In (E) and (G–I), nuclear envelope is blue, and *Wolbachia* and host DNA are red (OliGreen). White arrows indicate *Wolbachia*. (A) A stage 7 oocyte derived from a hs-Dmn/+ female, heat shocked. *Wolbachia* localize normally in the anterior region. (B) A stage 7 oocyte derived from a hs-Dmn/hs-Dmn female, heat shocked. *Wolbachia* become displaced from the anterior region. (C) A series of early-stage infected egg chambers. Dynein becomes enriched in the oocyte before stage 7. (D) A stage 5–6 infected oocyte. Within the oocyte, Dynein is enriched near the posterior pole and in a ring around the periphery of the oocyte nucleus. Many *Wolbachia* localize near nuclear-associated Dynein along the anterior periphery of the oocyte nucleus but not with the majority of Dynein concentrated at the posterior pole. Scale bar = 20 μm. (E) A stage 5–6 infected oocyte. In the oocyte, many *Wolbachia* maintain a close association with the nuclear envelope. (F) A stage 9 infected oocyte. Following the microtubule reorganization, Dynein localizes at the posterior pole of the oocyte and between the oocyte nucleus and the anterolateral cortex. At this time, *Wolbachia* become distributed throughout the oocyte cytoplasm. Scale bar = 20 μm. (G) A stage 9 infected oocyte. Following the microtubule reorganization, *Wolbachia* lose their association with the nuclear membrane. (H) A stage 6 *Dhc64C* ^6–6^/*Dhc64C* ^6–12^-derived oocyte. *Wolbachia* localize around the entire periphery of the oocyte nuclear envelope. (I) A stage 6 *Dhc64C* ^6–6^/*Dhc64C* ^6–12^-derived oocyte. *Wolbachia* dissociate from the oocyte nuclear envelope.

In addition to morphogen transport in the oocyte, cytoplasmic Dynein is a known transporter of membranous vesicles and organelles in many different cell types, and is tethered to these cargoes by Dynactin, a large, multi-subunit complex [50,51]. To ascertain whether the Dynein-dependent localization of *Wolbachia* also relies on Dynactin, we analyzed the bacteria in oocytes in which the p50/Dynamitin (Dmn) subunit of Dynactin was overexpressed using a heat-shock-driven promoter. It has been well established that overexpression of Dmn disrupts the association of Dynein with its cargo in both tissue culture cells and in the *D. melanogaster* germ line [52,53]. In oocytes from females carrying a single copy of hsDmn, *Wolbachia* localized normally near the anterior cortex (Figure 8A). However, in all of 16 stage 4–7 oocytes derived from females carrying two copies of hs-Dmn, *Wolbachia* were mislocalized to varying degrees within the oocyte cytoplasm (Figure 8B). This result further supports the idea that normal bacterial localization requires Dynein, and suggests that an interaction between *Wolbachia* and the Dynein complex is mediated through Dynactin.

### *Wolbachia* Localize Near the Anterior Nuclear Envelope of the Oocyte

To visualize *Wolbachia* relative to cytoplasmic Dynein in the oocyte, we stained infected egg chambers with antibodies raised against the Dhc. In stage 1–7 egg chambers Dynein exhibits a preferential accumulation within the oocyte (Figure 8C) [54]. During this time, Dynein concentrates at a position between the oocyte nucleus and the posterior cortex thought to represent the MTOC, and additionally in a ring surrounding the periphery of the oocyte nucleus (Figure 8D) [54,55]. Staining with antibodies against Lamin Dm0 confirmed that this ring of Dynein is associated with the surface of the nuclear envelope (Figure 8E). At this time, many *Wolbachia* in the oocyte localized along the anterior side of the nuclear membrane and near the ring of nuclear-membrane-associated Dynein, but the majority of bacteria did not appear to co-localize with Dynein in this region. Additionally, in oocytes with larger numbers of *Wolbachia,* the bacteria fully occupied the space between the nuclear envelope and the anterior cortex. However, in oocytes with fewer *Wolbachia,* the bacteria maintained a closer association with the oocyte nuclear envelope and did not appear to associate with the anterior cortex (not shown).

Following the microtubule rearrangement, the oocyte nucleus migrates to a corner of the anterolateral cortex. At this time, Dynein localized between the oocyte nucleus and cortex and in a cap at the posterior pole (Figure 8F) [54], while *Wolbachia* were distributed evenly throughout the oocyte cytoplasm (Figure 8F). Although many *Wolbachia* localized near the anterior nuclear envelope between stages 3–7 (Figure 8E), the bacteria completely dissociated from the anterior region and the nuclear envelope and became distributed throughout the cytoplasm (Figure 8G).

To test whether Dynein is required for positioning of *Wolbachia* along the anterior side of the nuclear envelope, we stained the bacteria and the nuclear envelope in oocytes derived from *Dhc64C* ^6–6^/*Dhc64C* ^6–12^ females. In these oocytes, *Wolbachia* were either misplaced around the entire nuclear envelope (Figure 8H), or they dissociated partially or completely from the nuclear envelope (Figure 8I). Taken together, these results suggest that Dynein is required not only for localization of *Wolbachia* near the nuclear envelope, but also for positioning the bacteria specifically at its anterior side.

### Integrity of the Microtubule Network and Cytoplasmic Dynein Are Required for Normal *Wolbachia* Numbers in the Oocyte

To determine whether microtubules play a role in *Wolbachia* levels in addition to their localization, we counted total *Wolbachia* numbers in normal and colchicine-treated stage 4–5 oocytes and nurse cells (see Figure 4B). Control oocytes and nurse cells exhibited bacterial means of 127 (standard error of the mean, ± 12) and 52 (± 3), respectively, while colchicine-treated oocytes and nurse cells exhibited bacterial means of 59 (± 13) and 76 (± 7), respectively. *Wolbachia* numbers were significantly higher in control oocytes than in nurse cells (*t*-test, *p* < 0.001, degrees of freedom [df] = 56), whereas bacterial numbers in treated oocytes were reduced to levels not statistically different from nurse cells (*p* = 0.248, df = 38). Additionally, bacterial numbers were significantly lower in treated oocytes than control oocytes (*p* < 0.001, df = 47), although bacterial numbers for treated nurse cells were higher than for control nurse cells (*p* = 0.001, df = 47).

These findings are further supported through genetic perturbation of the microtubule network in *maelstrom*-derived egg chambers (Figure 4C). *maelstrom* ^r20^/+ oocytes and nurse cells exhibited bacterial means of 118 (± 17) and 87 (± 12), respectively, while *maelstrom* ^r20^/*Df (3L) 79E-F* oocytes and nurse cells exhibited bacterial means of 65 (± 8) and 70 (± 9), respectively. Within each genetic group, *Wolbachia* numbers in the oocyte were not significantly higher than *Wolbachia* numbers in the average nurse cell. However, there were significantly fewer bacteria in *maelstrom* ^r20^/*Df (3L) 79E-F* oocytes than in *maelstrom* ^r20^/+ oocytes (*p* = 0.007, df = 31), while bacterial numbers in nurse cells of each group did not differ (*p* = 0.235, df = 31). These results, taken together with results from colchicine treatment, suggest that disrupting the microtubule network significantly decreases the number of *Wolbachia* in the oocyte.

To discern whether manipulation of Dynein also affects *Wolbachia* numbers in the oocyte, we obtained total bacterial numbers for oocytes and nurse cells derived from *Dhc64C* ^6–6^/+, *Dhc64C* ^6–12^/+, and *Dhc64C* ^6–6^/*Dhc64C* ^6–12^ individuals (Figure 4D). The mean number of *Wolbachia* in *Dhc64C* ^6–6^/+, *Dhc64C* ^6–12^/+, and *Dhc64C* ^6–6^/*Dhc64C* ^6–12^ oocytes was 203 (± 29), 155 (± 22), and 140 (± 27), respectively, while the means for corresponding nurse cells were 89 (± 15), 67 (± 8), and 106 (± 12), respectively. Despite the fact that bacterial numbers in *Dhc64C* ^6–6^/*Dhc64C* ^6–12^ oocytes appeared lower than in control (heterozygous) oocytes, Two-way ANOVA showed no significant differences in bacterial number in oocytes or nurse cells across these three genetic groups (F_2,86_ = 1.48, *p* = 0.233). In all cases, oocyte bacterial numbers remained significantly higher than nurse cell numbers (F_1,86_ = 22.04, *p* < 0.0001).

## Discussion

### *Wolbachia* Exhibit a Distinct Anterior Localization in the Oocyte

By characterizing the distribution of *Wolbachia* in the female germ line of *D. melanogaster,* we have discovered that these endoparasitic bacteria accumulate anteriorly in the oocyte during mid-oogenesis. In this regard, *Wolbachia* are reminiscent of several embryonic axis determinants, including *oskar, bicoid,* and *gurken* mRNAs, which also exhibit distinct localization patterns in the oocyte. These axis determinants must be positioned at specific regions within the oocyte at different stages for proper embryonic development. For example, *oskar* localizes in a tight band at the posterior cortex from stage 9 through the remainder of oogenesis, where it is required for formation of the germ plasm [56,57]. Like *Wolbachia, bicoid* also localizes anteriorly [58,59]. However, *Wolbachia* localize anteriorly from stage 3 to 7, while *bicoid* localizes anteriorly from stage 8 through the remainder of oogenesis. *gurken,* required for establishment of the dorsal–ventral axis, accumulates along the posterior side of the nucleus between stages 1 and 7 and then between the nucleus and anterolateral cortex at stage 8 [60,61]. Similarly, *Wolbachia* appear to associate transiently with the nuclear membrane, but on its anterior side and at stages preceding *gurken* nuclear localization. Although the significance of *Wolbachia*'s anterior concentration is not known, the fact that it does not overlap with key developmental determinants may have evolved to diminish the likelihood of the bacteria disrupting proper oocyte development.

### *Wolbachia* Behavior Requires Host Microtubules in the Oocyte and Nurse Cells

Our cytology has shown that *Wolbachia* localization in the egg chamber is microtubule dependent. In the oocyte, *Wolbachia* normally lose their anterior localization and become cytoplasmically distributed following the programmed reversal in polarity of the microtubule network between stages 7 and 8. Additionally, the bacteria are abnormally mislocalized in 90% of stage 4–6 colchicine-treated oocytes, in which microtubules are depolymerized, and in 60% of *maelstrom*-derived oocytes, in which normal positioning and polarity of the microtubule network is disrupted. Together, these data suggest that *Wolbachia* utilize the host microtubule network for their placement at the anterior pole of the oocyte. Interestingly, their localization is opposite the major MTOC of the egg chamber, which is positioned between the posterior cortex and the oocyte nucleus at this time. It is unlikely that *Wolbachia* localize near the anterior pole because they are excluded by a higher density of microtubules at the posterior region. Organelles such as endoplasmic reticulum and mitochondria are not excluded from the posterior pole. Endoplasmic reticulum is distributed evenly throughout the oocyte cytoplasm during early and middle stages [62], and mitochondria exhibit a slight enrichment near the posterior pole during these stages [39]. Additionally, *Wolbachia* do not aggregate at the oocyte's posterior pole following the microtubule reorganization, when microtubules are rich along the anterolateral cortex and in the surrounding cytoplasm and deficient near the posterior pole [63]. Therefore, it is likely that instead of being excluded from the posterior pole during stages 3–7, *Wolbachia* use microtubules for specific placement at the anterior region of the oocyte.

The dependence of *Wolbachia* on microtubules for localization in the oocyte is consistent with their close association with the astral microtubules of centrosomes in the early *Drosophila* embryo [4,5]. The fact that *Wolbachia* require host microtubules for proper localization both in the oocyte and in the embryo suggests that the microtubules are the primary cytoskeletal scaffold used by *Wolbachia* for germ-line and somatic transmission. Additional support for this idea comes from our finding that the filamentous Actin depolymerizer, cytochalasin-D, does not disrupt *Wolbachia* localization in the oocyte.

### *Wolbachia* Require the Dynein Complex for Localization in the Oocyte

Our mutational analysis has shown that *Wolbachia* localization is disrupted in oocytes from *Dhc64C* ^6–6^/*Dhc64C* ^6–12^ females and in oocytes in which the Dmn subunit of Dynactin is overexpressed. These observations suggest that in the oocyte, *Wolbachia* rely on the Dynein complex for association with microtubules. Dynein plays multiple critical roles in oogenesis. Although the mechanisms are not clear, Dynein is involved in the early localization of maternal factors for establishment and maintenance of oocyte identity [54,64], the migration and anchoring of the oocyte nucleus to the plasma membrane [53,55], and the localization of specific axis determinants to their proper positions within the oocyte [53,55,59].

Despite the fact that *Wolbachia* require Dynein for proper behavior in the early oocyte, the bacteria do not co-localize with the highest concentrations of this motor complex. How then do these bacteria depend on Dynein and microtubules for their subcellular behavior? One idea is that *Wolbachia* use Dynein as a transporter. Dynein is believed to be a major transporter of axis determinants into the oocyte from nurse cells [54,64]. Conceivably, a subset of *Wolbachia* may use Dynein to move along microtubules through ring canals into the oocyte [16]. As the bacteria enter the oocyte at its anterior region, they may dissociate from Dynein and accumulate there. At this time, *Wolbachia* may also associate with another host factor that anchors them to this region, thus inhibiting their movement. This idea is supported by our observations that, in vivo, the bacteria exhibit movement in nurse cells, whereas in the oocyte they are relatively motionless. Following microtubule reorganization, the bacteria lose their affinity for the anterior region and become distributed throughout the oocyte cytoplasm, where they persist for the remainder of oogenesis. The fact that *Wolbachia* accumulate neither along the anterolateral cortex—where microtubule minus ends are located—nor at the posterior pole—where Dynein accumulates as a consequence of Kinesin-1-mediated recycling—supports the idea that the interaction between *Wolbachia* and Dynein is transient. Our data do not allow us to determine precisely how *Wolbachia* localize at the anterior pole of the oocyte during early stages. Nevertheless, their accumulation there suggests a novel quality of this region of the oocyte that will be of interest to discern in future studies.

Genetic manipulation of Dynein resulted in mislocalization of *Wolbachia* but did not decrease their numbers in the oocyte. This is consistent with the nature of the *Dhc* alleles used here, which resulted specifically in misplacement of Dynein [49]. Stronger alleles that reduce Dynein levels result in failure to form an oocyte [65] and, therefore, could not be used to test the idea of Dynein as a transporter of *Wolbachia*. Improvements in other methods such as live bacterial imaging will be important for further testing of this idea.

We have shown that *Wolbachia* numbers increase in nurse cells and in the oocyte in stages 2 through 7, indicating that bacterial replication occurs in both cell types during this time. However, although the volumes of both cell types increase at similar rates during this period, *Wolbachia* increase by approximately 160% in the average nurse cell, in contrast to approximately 230% in the oocyte. In addition to transport from nurse cells, it is possible that higher bacterial replication rates in the oocyte contribute to this difference. *Wolbachia* replication in the oocyte may be enhanced by the distinct subcellular environment of this cell, including a higher concentration of microtubules and the oocyte's accumulation of developmental determinants, organelles, and nutrients. Previous studies have shown that *Wolbachia* are enclosed within a layer of host-derived membrane [4,66]. Compared to nurse cells, the oocyte is enriched with endoplasmic reticulum [62], which could serve as a source of host membrane for the bacteria. By associating with microtubules through Dynein, *Wolbachia* may intercept membrane vesicles or other host factors trafficked along microtubules, thus facilitating its own replication. This idea is supported by previous findings that other bacterial endoparasites, such as *Salmonella typhimurium,* require host microtubules and Dynein for replication [67,68].

### *Wolbachia* and Mitochondria Exhibit Different Localizations

Mitochondria are bacterial in origin and, like *Wolbachia,* are maternally transmitted. In addition, *Wolbachia*'s outer layer of host-derived membrane may allow them to escape the host immune response by mimicking organelles, such as mitochondria. Therefore, a plausible hypothesis is that *Wolbachia* rely on mechanisms similar to mitochondria for transmission through the female germ line. Although mitochondria exhibit a dependence on microtubules for their localization in tissues of many higher eukaryotes [69–71], their distribution differs markedly from *Wolbachia* during oogenesis. During formation of the germ cells, a subset of mitochondria and other organelles such as Golgi aggregate into a cloud-like structure known as the Balbiani body, while the rest remain cytoplasmic [39]. While mitochondria aggregate along the branched arms of the fusome at the center of the cyst and within the Balbiani body during germ cell division [39], *Wolbachia* remain distributed throughout the germ cell cytoplasm. Between stages 1 and 7, mitochondria either move to the posterior pole of the oocyte to associate with the germ plasm or remain distributed within the cytopolasm [39], in contrast to *Wolbachia,* which localize near the anterior pole (see Figure 2). Based on their different patterns of localization, it is likely that *Wolbachia* and mitochondria have evolved different mechanisms for associating with microtubules in the female germ line.

### *Wolbachia* Infect the Oocyte during Early and Late Phases

Our observations suggest that *Wolbachia* infect the oocyte at two distinct periods during oogenesis. First, *Wolbachia* become distributed among all of the germ cells as they form in region 1 of the germarium. This provides a means for the bacteria to establish infection in the oocyte very early in development, allowing them to multiply in the oocyte and 15 nurse cells until the time when cytoplasmic dumping occurs. Beginning at stage 10, this process results in the transfer of all nurse cell cytoplasm into the oocyte. Accurate bacterial counts for oocytes later than stage 7 were unobtainable owing to large cell size and yolkiness of cytoplasm. However, based on bacterial counts from stage 6–7 egg chambers, we estimate that approximately 6-fold more bacteria are delivered into the oocyte from nurse cells than are present in the oocyte before cytoplasmic dumping. Therefore, this second phase of bacterial transfer explains how the majority of *Wolbachia* become transmitted into the egg.

Infrequently we observed middle stage uninfected oocytes within infected egg chambers, which could result from imperfect bacterial distribution during division of the germ cells. However, by allowing for a high level of bacterial magnification and a second source of bacterial transfer into the oocyte, the nurse cells are likely to insure that these uninfected oocytes will eventually become infected. Bacterial amplification in nurse cells and subsequent transfer into the oocyte might therefore underlie the near-perfect transmission of infection observed in some *Drosophila* species [24,72]. The idea that bacterial amplification in nurse cells is important for infection of the oocyte has been suggested previously for other insect species [20,73].

### Conclusions

While the interactions between intracellular pathogens and the host Actin cytoskeleton have been intensively investigated [3], those between pathogens and the microtubule cytoskeleton are less well explored. The *Wolbachia*–*Drosophila* system provides powerful tools for dissecting the complex nature of such parasite–host interactions, allowing the identification of specific host factors exploited by these intracellular bacteria. The results presented here demonstrate that *Wolbachia* utilize the host microtubule network for their subcellular localization in the *Drosophila* oocyte. These interactions may also play a role in bacterial motility and replication, ultimately leading to the bacteria's efficient maternal transmission. Our identification of cytoplasmic Dynein and Dynactin as potential links between *Wolbachia* and microtubules illustrates the promise of this system in molecularly defining these interactions through genetic means. Studies of the mechanisms by which pathogens commandeer the Actin cytoskeleton have proven useful in elucidating basic host mechanisms of Actin polymerization [2]. Likewise, the discovery of *Wolbachia*'s striking anterior localization in the *Drosophila* oocyte, which is distinct from the localization of mitochondria and all known morphogens, indicates that the analysis of *Wolbachia*–host interactions also may inform us about basic host cellular and developmental processes.

## Materials and Methods

### Fly strains and establishment of infection.

Two *D. melanogaster* lines (both OregonR-derived) infected with the *w*Mel *Wolbachia* strain were used for bacterial characterization in wild-type egg chambers. A *D. melanogaster white* ^1118^ line infected with *w*Melpop (Popcorn) [43] and a *D. simulans* line infected with *w*Ri [42] were also used. *w*Mel from the OregonR line was introgressed into a *CyO*/*Sco* line, which was then used to infect the following lines for mutational analysis: *Dhc64C* ^6–6^ and *Dhc64C* ^6–12^ [65], *maelstrom* ^r20^ and *Df (3L) 79E-F* [48], *P(w+, FRT}42B Khc* ^27^ [74] and hs-Dmn [53]. Germ-line clones of *Khc* ^27^ were generated as described in [74]. Overexpression of Dmn was carried out as described in [53]. The infection status of each line was confirmed by PCR of the *Wolbachia* 16S rRNA or *Wolbachia* Surface Protein genes as described in [75].

### Cytoskeletal inhibitor treatment.

For disruption of microtubules, 3- to 5-d-old female flies were starved for 18 h and then fed yeast paste supplemented with 50 μg/ml colchicine for 24–30 h before ovaries were dissected in EBR solution [44]. For depolymerization of filamentious Actin, ovaries were dissected and soaked in EBR solution containing 20 mg/ml cytochalasin-D for 20 min [63].

### Analysis of *Wolbachia* in vivo.

Ovaries were dissected from 2- to 3-d-old females and incubated for 20 min in 5 mM Draq 5 (Alexis Biochemicals, Montreal, Quebec, Canada). Ovaries were transferred to halocarbon oil, where individual ovarioles were separated for imaging. Egg chambers were imaged with the PerkinElmer (Wellesley, Massachusetts, United States) Ultra View RS spinning disk confocal system. Images were collected at 15-s intervals and real time was accelerated 75 times.

### Fixation, immunostaining, and fluorescence microscopy.

Following dissection, ovaries were fixed for 10 min in 3.7% paraformaldehyde/heptane [76]. Primary antibodies were incubated overnight at 4 °C at the following dilutions: rabbit anti-α-Spectrin at 1:150 [77], mouse anti-α−Tubulin at 1:150 (Sigma, St. Louis, Missouri, United States), mouse anti-Lamin Dm0 at 1:50 [78], and mouse anti-Dhc at 1:200 [54]. Following six 15-min washes in PBS with 0.1% Triton-X-100, ovaries were incubated at room temperature for 1 h with Alexa 488– or Alexa 633–coupled anti-mouse antibodies (1:150) (Molecular Probes, Eugene, Oregon, United States) and/or FITC-conjugated phalloidin (1:100) (Molecular Probes). *Wolbachia* were stained with anti-hsp60 (1:500) (Sigma), OliGreen (1:750) (Molecular Probes), DAPI (Molecular Probes), or propidium iodide added to the mounting medium. In experiments in which *Wolbachia* were visualized with DNA dyes, fixed egg chambers were RNaseA-treated for 6–8 h at 37 °C before staining. Images were generated using a Leica (Wetzlar, Germany) DM IRB confocal microscope and processed using Adobe Photoshop 7.0 (Adobe Systems, San Jose, California, United States). Bacterial counts were obtained from serial sections taken at 1-μm intervals through entire oocytes and nurse cells. Statistical analyses were performed using SYSTAT, version 10.2 (Systat Software, Point Richmond, California, United States).

## Supporting Information

Video S1 *Wolbachia* Exhibit Movement in the Nurse Cells but Not in the Oocyte In Vivo (1.2 MB ZIP)

Video S2 The DNA Stain Draq 5 Highlights Only Host DNA in Live Uninfected Egg Chambers (805 KB ZIP)

### Accession Numbers

The NCBI Entrez (http://www.ncbi.nlm.nih.gov/gquery/gquery.fcgi?itool=toolbar) accession numbers for the genes and gene products discussed in this paper are cDNA Dmn subunit of Dynactin (AY061092), Dhc (P37276), human hsp60 (AAF66640), Khc (P17210), Lamin Dm0 (P08928), *maelstrom* (AAB97831), and α-Spectrin (AAB29441).

## Abbreviations

df: degrees of freedom
Dhc: Dynein heavy chain
Dmn: p50/Dynamitin
hsp60: heat shock protein 60
Khc: Kinesin heavy chain
MTOC: microtubule-organizing center
